# The structural and content aspects of abstracts versus bodies of full text journal articles are different

**DOI:** 10.1186/1471-2105-11-492

**Published:** 2010-09-29

**Authors:** K Bretonnel Cohen, Helen L Johnson, Karin Verspoor, Christophe Roeder, Lawrence E Hunter

**Affiliations:** 1 Department of Pharmacology, Center for Computational Pharmacology, University of Colorado School of Medicine, Aurora, Colorado, USA; 2 Department of Linguistics, University of Colorado at Boulder, Boulder, Colorado, USA

## Abstract

**Background:**

An increase in work on the full text of journal articles and the growth of PubMedCentral have the opportunity to create a major paradigm shift in how biomedical text mining is done. However, until now there has been no comprehensive characterization of how the bodies of full text journal articles differ from the abstracts that until now have been the subject of most biomedical text mining research.

**Results:**

We examined the structural and linguistic aspects of abstracts and bodies of full text articles, the performance of text mining tools on both, and the distribution of a variety of semantic classes of named entities between them. We found marked structural differences, with longer sentences in the article bodies and much heavier use of parenthesized material in the bodies than in the abstracts. We found content differences with respect to linguistic features. Three out of four of the linguistic features that we examined were statistically significantly differently distributed between the two genres. We also found content differences with respect to the distribution of semantic features. There were significantly different densities per thousand words for three out of four semantic classes, and clear differences in the extent to which they appeared in the two genres. With respect to the performance of text mining tools, we found that a mutation finder performed equally well in both genres, but that a wide variety of gene mention systems performed much worse on article bodies than they did on abstracts. POS tagging was also more accurate in abstracts than in article bodies.

**Conclusions:**

Aspects of structure and content differ markedly between article abstracts and article bodies. A number of these differences may pose problems as the text mining field moves more into the area of processing full-text articles. However, these differences also present a number of opportunities for the extraction of data types, particularly that found in parenthesized text, that is present in article bodies but not in article abstracts.

## Background

Two exciting developments in recent years have the potential to change the face of biomedical natural language processing or text mining (BioNLP). One of these is the attention that the community has begun to turn to using full-text journal articles as inputs--previously (and continuing into the present), the majority of BioNLP work had been done with abstracts as input. The other development is the birth and growth of PubMedCentral (PMC). PMC is a repository for full-text journal articles, both Open Access and traditionally copyrighted. As of 2009 it contains 1.3 million full-text (FT) journal articles, and is growing rapidly, creating an unprecedented opportunity for text mining.

There has been an awareness since the early days of the modern era in BioNLP that there are differences between the content of the abstracts and the content of the bodies of journal articles. Various authors (see below) have described particular aspects of these differences. However, no one has yet attempted a broad characterization of the differences between them. Structural differences between them also have not been addressed. This article does just that, with an emphasis on those aspects of the content and structure of abstracts and FT bodies that have implications for text mining. With that focus, this article can be seen as not only descriptive of the differences and similarities between FT and abstracts, but as suggesting a roadmap for developing the language processing tools and infrastructure that will be necessary to fully exploit the new data available to us in PMC.

As our input, we primarily use a corpus called CRAFT, the Colorado Richly Annotated Full Text corpus [1]. CRAFT consists of 97 full-text journal articles, i.e. including both the abstract and the body of the article. (We will use the term *article *to refer to the entire contents of the article, i.e. including both the abstract and the body; *abstract *to refer to the title and abstract; and *body *to refer to the contents minus the title and abstract. We will also sometimes use the term *body *to refer to the contents minus the title, abstract, and the bibliography; we will make explicit to which we are referring below.) To ensure biological relevance, the contents of CRAFT were selected in cooperation with the Mouse Genome Informatics database. All articles are relevant to mouse genomics and have been curated by MGI personnel, and they are all the basis for at least one Gene Ontology gene annotation in the MGI database. In an ongoing three-year project expected to be completed in 2010, we are carrying out extensive linguistic and semantic annotation of the CRAFT data. This includes annotation of linguistic features such as full syntactic parse trees and semantic categories such as gene names and Gene Ontology terms. All articles were available in the PubMedCentral XML format, which segments an article into its abstract and body, and within the body segments the introduction, materials and methods section, results, discussion, acknowledgements, bibliography, etc. This segmentation allows us to compare the abstract versus the body of the 97 articles in CRAFT.

Our goal was to compare abstracts and bodies to features that are relevant to language processing. Out of the many such features that may exist, we picked a number that were both amenable to automatic assessment and that could lead to actionable information on how to advance the state of the art. We also used some of the manually annotated data for these assessments. We detail below the specific features that we examined. Briefly, they included (a) structural aspects of the abstracts and bodies; (b) morphosyntactic and discourse features of the contents; (c) distributions of semantic classes of named entities; and (d) differential performance of text mining tools.

### Related work

Various authors have looked at general distributional differences of semantic content between abstracts and article bodies [2] looked at the case of protein-protein interactions and concluded that high coverage could not be achieved without processing article bodies [3] found that biologically relevant words are more dense in abstracts, but that there are many more of them in article bodies [4] found that more than half of the information on protein-protein interactions in articles is found in the body. Similarly, [5] examined a single article and found that only seven of nineteen unique interactions in the paper were mentioned in the abstract, with the rest occurring only in the full text.

Other authors have investigated other aspects of full text versus abstracts [6] looked at the effectiveness of searching full text versus abstracts for information retrieval purposes and found that searching full text was more effective as measured by MAP, P20, and IPR50, especially when spans of the full-text articles were considered [7] downsampled 2,162 sentences from 78 full-text articles and annotated them for a variety of factors that affect coherence phenomena. They found that coreference is very important, as is dependency between separate sentences [8] note that sentences found in Introduction, Methods, Results, and Discussion sections are not necessarily the type that would be expected in those sections. For example, they found "Methods"-type sentences in a Results section. They annotated sentences in full-text documents as to which section type they actually represented and trained a classifier to accurately label sentence types. Crucially, they found that a classifier which was trained to perform the same task on abstracts did not perform well when applied to full text.

Still other authors have pointed out that there are challenges posed by full-text journal articles that are not an issue with abstracts. For example, [9] and many others since have noted problems in dealing with non-ASCII characters, particularly Greek letters. Full text articles can also present other challenges, such as the recognition and clean-up of embedded tags, non-ASCII characters, tables and figures, and even the need to convert from PDF to textual format. Access to full text is an especially troublesome issue.

However, as these are not language processing problems per se, they are not addressed further in this work. Other relevant work has dealt with the processing of full-text articles in general. Early work by Friedman and Rzhetsky [5] led to the development of the GENIES system, which uses both semantic and syntactic grammars to process full-text articles. More recently, Rzhetsky's GeneWays system has also dealt with full text, analyzing nearly 150,000 FT articles and using the results to populate a database of almost 1.5 million statements about signalling pathways at a precision of 95%. In the very recent past, the TREC Genomics [10] and BioCreative competitions [11,12] have led to a large increase in work on full-text articles.

## Results and Discussion

We examined the following:

Structural aspects:

• Distribution of sentence lengths

• Incidence and types of parenthesized text

Morphosyntactic and discourse features:

• Incidence of coordination, negation, passives, and pronominal anaphora

• Distributions of semantic classes of named entities:

• Distribution of gene/protein names

• Distribution of mutations

• Distribution of drug names

• Distribution of diseases

Differential performance of text mining tools:

• Gene mention performance

• Mutation mention performance

## Structural aspects

### Distribution of sentence lengths

Sentence length has a consequence for natural language processing: the performance of full syntactic constituency parsers as evaluated on the sentence level (as opposed to the constituent level) is poor for long sentences [13]:480. For this reason, if article bodies have notably longer sentences than do abstracts, we will need either more and better training data for them, or even a different approach than traditional constituent structure parsers, such as dependency parsing. On the other hand, if article bodies have notably shorter sentence lengths than do abstracts, then we can expect them to be easier to parse than abstracts. To test whether there is a difference between sentence length distributions in article abstracts and bodies, we used a sentence segmenter (see Methods) to split each section into individual sentences. We then tokenized each sentence on white space. (This yields the traditional segmentation for word-counting in corpus linguistics.) Sentence length was then determined in words for each sentence in the corpus.

Table 1 shows the resulting median and mean sentence lengths for each section of the article and shows the results of comparisons between all article sections. Mean sentence length distributions were statistically significantly different between the abstract (in this section, "abstract" means just the abstract, exclusively of the titles, since titles are not generally sentences) and all sections of the article body at P <.01 for all as determined by Mann-Whiteney-Wilcox signed rank test, with Bonferroni correction for multiple comparisons. MWW was used since the distribution of sentence lengths is not normal. Crucially, sentence length means were shorter for the abstract than for all other sections except for captions. Median lengths were shorter for all sections but the Methods and Conclusions, as well. This suggests that full parsing of article bodies may be more difficult than full parsing of abstracts. We also discuss the implications of this finding for overall sentence complexity below.

**Table 1:** Comparison of sentence length between different article sections.

	**Abst**.	**Intro**.	Results	**Meth**.	**Disc**.	**Concl**.	**Capt**.
Abst.							

Intro.	*						

Results	*	*					

Methods	*	*	*				

Disc.	*	--	--	*			

Concl.	--	--	--	--	--		

Capt.	*	*	*	*	*	*	

Median/mean	25/26.49	28/29.52	29/31.17	23/26.80	29/30.35	26/28.34	21/24.85

Statistically significantly different pairs at P < .01 are marked by *. Pairs that are not statistically significantly different are marked by --.

### Incidence and types of parenthesized text

We looked at the incidence and types of parenthesized text for two reasons. One is that parenthesized text is out of the scope of normal syntactic rules, and therefore can be expected to pose a challenge for syntactic parsers. The other is that different types of syntactic text require (or present opportunities for) different kinds of handling by information extraction and other applications. For example:

• Parenthesized gene symbols and abbreviations allow for improved coreference resolution within a text, as well as gene normalization.

• Parenthesized data might be a target for information extraction applications [14], [15].

• Parenthesized P values might be valuable for the automated extraction of meta-analyses.

• Parenthetical *text*, by definition, may be completely ignorable.

• Parenthesized table and figure captions are often indicators of assertions with experimental validation [15].

• Parenthesized citations are useful for establishing rhetorical relations between papers [16], synonym identification [14], and curation data [14].

Thus, we are interested in the overall frequencies of parenthesized text in abstracts and article bodies, and are also interested in the distribution of types of parenthesized material between the two. (In interesting work on adapting medical text for lay readers, [17] pointed out that parenthesized material in technical text is almost always distracting and incomprehensible to the lay reader. However, looking at her examples, it is clear that this information is highly relevant to the scientifically trained reader's interpretation of the text, and could profitably be text mined.)

**Figure 1:**
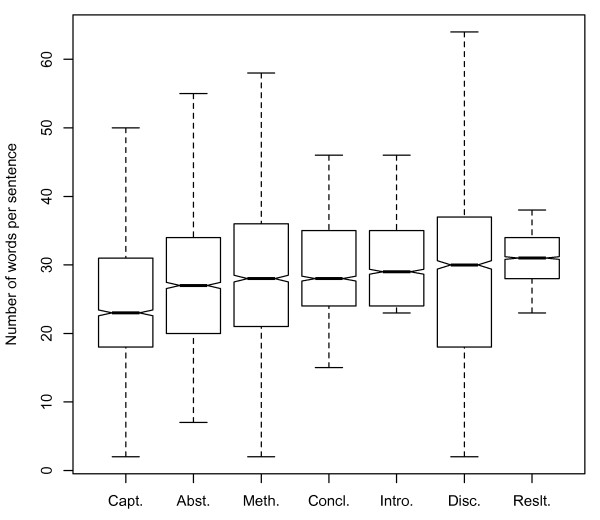
**Distribution of sentence lengths across the various article sections**. Distribution of mean sentence lengths across the various article sections. See Table 1 for significance.

To test whether there is a notable difference between abstracts and article bodies with respect to parenthesized materials, we wrote an application that counted parenthesized text strings and attempted to classify them, using regular expressions, into one of nine categories (Java and Perl versions of the application are available at http://bionlp.sourceforge.net):

• Abbreviations or gene symbols (e.g. *We mapped two quantitative trait loci (QTLs)*)

• Citations (e.g. *Fambrough et al. 1999*)

• Data values (e.g. *h2 = 0.39*)

• P-values (e.g. *P < .001*)

• Parenthetical statements (e.g. *Bsc10a maps to the central region of Chr 10 (LRS of 17.5 near D10Mit186)*)

• Figure/table pointers (e.g. Fig 1A)

• Singular/plural markers (e.g. *the s in gene(s)*)

• Parts of gene names (e.g. *NF-kappa(b)*)

• List elements (e.g. *brains weight (A), striatal neuron-packing density (B), and striatal neuron number (C)*)

• "unknown"

For those categories for which we could do a principled assessment of the correctness of the classification, we manually examined the results of the classification. We found accuracies ranging from a low of 76% (for data values) to a high of 100% (for citations). We point out that our evaluation was very stringent, so that e.g. in the case of the data value category, if the parenthesized text contained data values but also contained a P-value, we marked it as incorrect. The number of instances of parenthesized materials that the script could not classify was not small--26.99% for the abstracts and 28.69% for the article bodies--so the number of instances of each category that we report is probably an underestimate; the recall of the script is apparently about 75%, modulo any instances that might not fit into one of these nine categories, such as mixed data and P-values.

We found marked differences both in the incidence of parenthesization and in the distribution of types of parenthesized materials between the abstracts and the article bodies. These are summarized in Table 2. Regarding incidence, parenthesis usage in the article bodies was almost triple that in the abstracts, with 28 per thousand words in the bodies and only 10 per thousand words in the abstracts. The distribution of types was different as well, with abbreviations and gene symbols accounting for over half of the uses of parentheses in the abstracts (at least 55%), but only 11% of the uses of parentheses in the article bodies. There was also a smaller diversity of uses of parentheses overall in the abstracts. Only six different types of parenthesized material were seen in the abstracts:

**Table 2:** Summary of results on parenthesis usage distribution.

	Abstracts	Bodies
List enumerators	3	1,399

Part of gene name	--	--

Table or figure	--	2,825

Citation	--	172

P value	2	146

Data	11	2,116

Singular/plural	2	33

Abbreviation or symbol	124	1,862

Parenthetical statement	23	3,453

Unknown	61	4,831

Total	226	16,837

list enumerators (3/226)

P-value (2/226)

data (11/226)

singular/plural (2/226)

abbreviation or gene symbol (124/226)

parenthetical statements (23/226)).

In contrast, eight of the nine uses of parentheses that we looked for were observed in the article bodies--all of the six that were seen in abstracts, plus tables and figures (2825/16,837), and citations (172/16,837). Handling parenthesized material may be a fruitful way to increase our yield of information from full-text articles.

## Morphosyntactic and discourse features

For significance testing of the data in this section, we used a two-sample two-tailed t-test on normally distributed data, and the Mann-Whitney-Wilcox signed-rank test on data that was not normally distributed. The results are summarized in Table 3.

**Table 3:** Differences in incidence of linguistic features per thousand tokens.

			Abstracts							Bodies		
**Tagger and model**	**P**	**R**	**F**	**TP**	**FP**	**FN**	**P**	**R**	**F**	**TP**	**FP**	**FN**

Human annotations				1180						22,432		

ABNER/BioCreative	0.634	0.393	0.485	464	267	716	.505	.322	.394	7,240	7,081	15,186

ABNER/NLPBA	0.629	0.363	0.460	429	253	751	.464	.298	.363	6,694	7,719	15,730

BANNER/BioCreative	0.678	0.482	0.563	569	270	611	.540	.473	.504	10,621	9,042	11,806

LingPipe/BioCreative	0.591	0.575	0.583	679	468	501	.353	.583	.440	13,094	23,992	9,330

LingPipe/GENIA	0.398	0.309	0.348	365	551	815	.250	.284	.266	6,380	19,101	16,046

LingPipe/JNLPBA	0.464	0.277	0.347	327	377	853	.289	.256	.271	5,741	10,498	16,684

Values marked with * are significantly different at the P < .01 level. Values marked with ** are significantly different at the P < .001 level.

### Incidence of coordination

The incidence of conjunctions was assessed because conjunctions are known to cause difficulties in parsing. As McClosky et al. put it, "Conjunctions are about the hardest things in parsing, and we have no grip on exactly what it takes to help parse them" (McClosky et al. 2006). We counted every instance of *and*, *or*, and *but *in the text, and calculated the ratio of coordinators to total tokens. The difference between abstracts and bodies was not statistically significant by two-sample two-tailed t-test, with only slightly more in article bodies--37.5 coordinations per thousand tokens, versus 36.2 coordinations per thousand tokens in abstracts.

### Incidence of passives

We looked at the incidence of passives since they pose some processing challenges for text mining, including the inversion of normal word order between agent and theme and the optionality of agents. To find passives, we looked for the text string *ed by*. This underestimates the incidence of passives, since it misses instances where there is no agent, and instances where there are multiple passive verbs in sequence (e.g. *Xed and Yed by*). However, the undercounting applies equally to the abstracts and to the article bodies, so the comparison between them remains valid. The incidence of passives was statistically significant between the two text types (p < .01) by the Mann-Whitney U Wilcox test, with more passives in the article bodies (4.3 per thousand tokens) than in the abstracts (3.7 per thousand tokens).

### Incidence of negation

We looked at the incidence of negation because like conjunction, handling negation is a perennial problem in natural language processing [18-21]. To detect negation, we looked for the text strings *no*, *not*, and *neither *in the text sets. The incidence of negation was statistically significant between the two text types (p < .01) by Mann-Whitney-Wilcox signed-rank test; again, the article bodies had a higher incidence at 5.3 per thousand tokens of text, versus 3.8 per thousand tokens of text in the abstracts.

### Incidence of pronominal anaphora

We looked at the incidence of pronominal anaphora since it gives an estimate of the importance of coreference resolution. Anaphoric reference has been cited as a cause of low recall in biological information extraction systems more often than would be suspected on the basis of studies that have actually looked at anaphora and coreference resolution in molecular biology texts [22,23]. We searched for pronouns by using a simple regular expression. This somewhat overestimates the incidence of *it*, since it does not differentiate between true pronominal uses and nonreferential uses (e.g. *It is clear that*...). However, again, the overestimate applies equally to the two text types, so the comparison remains valid. The incidence of pronouns is significantly different between the two text types (p < .001) by two-sample two-tailed t-test; abstracts had a modestly higher incidence, at 5.3 per thousand tokens of text versus 3.98 per thousand tokens of text in the article bodies.

### Sentence complexity

Having analyzed these various features of structure and content, we can ask a broader question: is there a difference in complexity between sentences in abstracts and in full text? Answering that question requires selecting a measure of sentence complexity. We examine here both measures of sentence complexity broadly construed, and of readability specifically. We note that the distinction between these is an unclear one, and that even the two notions in isolation are problematic. For example, when we ask if one sentence is more complex than another, we must assume some notion of what it is complex *for*, and surely this must be more than just complexity for a syntactic parser. Similarly, with respect to difficulty of reading, we must ask who is doing the reading and for what purpose.

Regarding syntactic complexity, [24] looked at a variety of linguistically motivated measures of sentence complexity and came to the conclusion that even sophisticated measures of complexity turned out to return the same results as a simple word count. Longer sentences are more complex than shorter sentences, and not even sophisticated measures give a better ordinal assignment of sentence complexity than that. We have already shown that there is a statistically significant difference in length between abstract sentences and body sentences--body sentences are significantly longer, and therefore more complex. However, Szmrecsányi's own Index of Syntactic Complexity did not reveal a difference in distributions of complexity between the two genres as evaluated by Kullback-Leibler divergence.

We also calculated a readability metric for both genres. Following the work of [25], we calculated the percentage of function words in sentences in the two genres and averaged it across the two text collections [25] found this percentage of function words to increase as readability increases. Interestingly, the readability metric shows no difference in readability between the two genres--abstracts have an average percentage of function words of 30.28 and bodies have an average percentage of function words of 30.35. Overall, these negative findings in the face of the statistically significant difference in sentence lengths between the two genres merit further investigation, but must remain the subject of future work, since it appears to reflect the power of the metrics themselves, rather than characteristics of article bodies versus abstracts. We discuss the implications for parsing below.

## Differential performance of text mining tools

### Gene mention tagger performance

**Figure 2:**
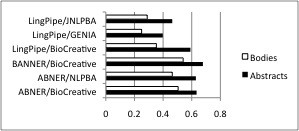
**Precision of the various tagger/model combinations**. Precision of the various tagger/model combinations. Each pair of bars shows the gene mention system followed by the model on which it was trained; e.g., LingPipe/JNLPBA is the LingPipe gene mention system trained on the JNLPBA data.

**Figure 3:**
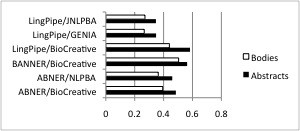
**F-measure of the various tagger/model combinations.** Recall of the various tagger/model combinations. F-measures of the various tagger/model combinations. Each pair of bars shows the gene mention system followed by the model on which it was trained; e.g., LingPipe/JNLPBA is the LingPipe gene mention system trained on the JNLPBA data.

**Figure 4:**
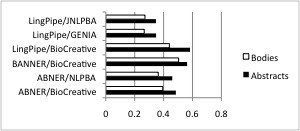
**Recall of the various tagger/model combinations.** Each pair of bars shows the gene mention system followed by the model on which it was trained; e.g., LingPipe/JNLPBA is the LingPipe gene mention system trained on the JNLPBA data.

Our corpus includes mark-up of all gene and protein mentions in 81 files of the corpus, roughly according to the BioCreative guidelines. We ran three gene mention systems (ABNER [26], BANNER [27], and LingPipe [28], chosen either for their popularity in the community or for their reported high performance) with up to three models apiece on the data. We found that for every tagger and every model, performance was higher on the abstracts than on the article bodies. Table 4 shows the precision, recall, TP, FP, and FN for each combination, and Figures 2, 3 and 4 show them graphically. F-measures were generally about 10 points higher on the abstracts than on the bodies, were never less than 6 points higher, and in one case was 14 points higher.

**Table 4:** Gene mention tagger performance for six combinations of tagger and model.

	Abstracts	Bodies
Conjunction	37.5	36.2

Passives	3.7*	4.3*

Negation	3.8*	5.3*

Pronominal anaphora	5.3**	3.98**

The first row gives the number of gene mentions in each set of texts.

### Mutation detection performance

To explore the performance of mutation detection in full text versus abstracts, we ran the MutationFinder [29] mutation detection system across both sections of the corpus. Mutations are not annotated in the corpus, so we ran the system and then examined its outputs manually. This allowed us to determine precision, but did not allow us to determine recall. Performance did not differ markedly. MutationFinder has previously been reported to have a precision of .984 on abstracts. We found it to have a precision of 1.00 on abstracts (although this may be misleading, since there were only two mutation mentions found in the 97 abstracts, probably making the published results a better comparison), and .959 on full text. Thus, performance differed somewhat, but is still quite high on the article bodies.

### Syntactic parser performance

To compare the performance of a constituent parser on abstracts versus bodies, we applied the Stanford Lexicalized Parser (v1.6) ([30]) to a subsection of the CRAFT corpus. The experimental corpus used to compare parser performance consisted of 37 abstracts (529 sentences, 10226 words) and 36 article bodies (8639 sentences, 216,860 words). (One article body had to removed from the evaluation because of a parsing anomaly.) We analyzed the resulting parses against the CRAFT gold-standard using EVALB (July 1, 2008 version), which implements the PARSEVAL metrics *bracket recall *and *tag accuracy *as described in [31].

Bracket recall is a reflection of the how well a parser has identified constituent boundaries in its candidate parses. Candidate constituents that are neither substrings nor superstrings of candidates in the gold-standard parse are called incompatible. Inversely, compatible candidate constituents are those that do not cross gold-standard constituent boundaries. (Single-word constituents are excluded from these definitions.) Bracket recall is calculated by dividing the number of compatible constituents in a candidate parse by the total number of constituents in the gold-standard parse. The mean bracket recall of the Stanford Parser on abstracts was 57%. On the article bodies the mean bracket recall was 59%. The difference was not statistically significant.

### Part of speech tagging

Tag accuracy is calculated by dividing the number of words tagged with the correct POS in the candidate parse by the total number of words in the sentence. On the abstracts Stanford's mean tag accuracy was 83%. On the body dataset, the mean tag accuracy was 81%. The better performance on the abstracts was statistically significant (p < .001) using the Mann-Whitney U Wilcox test.

## Distribution of named entity types

Table 5 shows the number of abstracts and article bodies mentioning the four semantic classes of named entities that we examined. Except for genes, the distribution of all semantic classes differed markedly. Note, for example, that 19 of 97 article bodies mentioned mutations, but only one abstract mentioned a mutation. Table 6 shows the average number of mentions of semantic classes in abstracts and article bodies. Not surprisingly, the average number of mentions was higher for all semantic classes in article bodies. Table 7 shows the density of mentions of each semantic class per thousand words in abstracts and article bodies.

**Table 5:** The number of abstracts and article bodies mentioning the four semantic classes of named entities that we examined, out of 97 abstracts and article bodies.

Semantic class	Abstracts mentioning	Bodies mentioning
Genes	94	97

Mutations	1	19

Drugs	18	85

Diseases	65	96

Except for genes, distributions differed markedly.

**Table 6:** Average number of mentions of semantic class in abstracts and bodies.

Semantic class	Abstracts average	Bodies average
Genes	15	280

Mutations	0.02	1.74

Drugs	0.72	13.6

Diseases	1	23

**Table 7:** Density of mentions of semantic class per thousand words in abstracts and bodies.

Semantic class	Abstracts	Bodies
Genes	61	47

Mutations	0.08*	0.28*

Drugs	2.97*	2.21*

Diseases	4.1*	3.74*

Statistically significantly different pairs at P < .01 are marked by *.

### Gene mentions

There was a distinct difference in distribution of gene mentions between abstracts and bodies, with an average of 15 gene mentions per abstract (1,180/80), and 280 per article body (22,432/81). This corresponded to a frequency of 61 gene mentions per thousand words in the abstracts ((1180*1000)/19,259) versus 47 gene mentions per thousand words ((22,432*1000/480,761))in the article bodies. However, genes were found both in abstracts and in bodies, with all bodies containing at least one gene mention and all but three abstracts containing at least one gene mention. The distributions of densities were not significantly different by the Mann-Whitney U Wilcox test. (It should be noted that this is a biased sample, since the corpus was selected such that at least one gene had to be mentioned in every article.)

### Mutation mentions

To explore the distribution of mutation mentions, we ran the MutationFinder mutation detection system, as described above. The distribution of mentions of mutations differed markedly between the two parts of the articles. Only one abstract contained any mutation mentions, while eighteen bodies contained a total of 169 mutation mentions, for an average of 1.74 mutation mentions per body but only 0.02 mutation mentions per abstract. The densities of mutation mentions were significantly different at P < .01 by the Mann-Whitney U Wilcox test, with densities of .08 ((2*1,000)/23,590) mutation mentions per thousand words in the abstracts versus .28 mutation mentions per thousand words ((169*1,000)/596,939) in article bodies.

### Drug mentions

To explore the distribution of drug mentions, we used a simple dictionary-based approach, which has been claimed to be adequate for drug names (Klinger, Kolarik, Fluck, Hofmann-Apitius, and Friedrich (2008)). As the dictionary, we used the DrugCards data from DrugBank (see the Methods section for details on the input file and on filtering of common English words, etc.). The distribution of disease mentions was quite different between the two parts of the articles--only 19/97 abstracts had drug mentions, but 85/97 bodies had drug mentions. The average number of drug mentions per paper was quite different in the abstracts and the bodies, with on average only 0.72 drug mentions per abstract but 13.6 drug mentions per body. The density of drug mentions was significantly different in abstracts and in bodies at P < .01 by the Mann-Whitney U Wilcox test, with 2.97 ((70*1,000)/23,590) drug mentions per thousand words in the abstracts and 2.21 ((1,322*1,000)/596,939) drug mentions per thousand words in the bodies.

### Disease mentions

To explore the distribution of disease mentions, we used the BANNER system. The distribution of disease mentions was quite different between the two parts of the articles. 32/97 abstracts had no disease mentions, but only a single article body lacked any disease mentions. This corresponded to an average of 1 disease mention per abstract and 23 mentions per body. The density of disease mentions was significantly different in abstracts and in bodies at P < .01 by the Mann-Whitney U Wilcox test, with 4.1 ((97*1000)/23,590) disease mentions per thousand words in the abstracts and 3.74 ((2,235*1000)/596,939) disease mentions per thousand words in the bodies.

### Implications of distributions and densities

A limitation of this paper is that we have not studied the effects of these distributions on any particular task, such as information extraction or information retrieval. However, there is evidence from the work of Lin [6] that searching full text is more effective than searching abstracts, especially when the search is restricted to text spans rather than full bodies.

## What is and is not normally distributed in biomedical journal articles?

This study allowed us to determine, for a number of categories of data, what is and is not normally distributed between abstracts and article bodies. We summarize this in Table 8.

**Table 8:** Phenomena that are and are not normally distributed between abstracts and article bodies.

Normally distributed	Not normally distributed
Coordination	Sentence length

Pronominal anaphora	Negation

	Passives

	Gene mentions

	Disease mentions

	Mutation mentions

	Drug mentions

Overall, more phenomena are not normally distributed than are normally distributed between the two genres.

## Conclusions

We found that abstracts and article bodies do differ from each other structurally and in terms of discourse features: article bodies have longer sentences and make much heavier use of parenthesized material of various sorts. Both of these findings have implications for the role of syntactic parsers in biomedical information extraction. The latter finding uncovers an opportunity for the extraction of various kinds of information from full text that is not available in abstracts. We also found that an important class of language processing system, gene mention systems, performs differently on articles and abstracts, with performance being notably higher on abstracts. Part of speech taggers also perform differently, with performance being better on abstracts. Distributions of mentions of most semantic classes differed markedly between abstracts and article bodies. A limitation of this study is that it is primarily descriptive. However, it does suggest some directions forward for full-text NLP. Overall, these findings suggest that to move forward with text mining from full text journal articles, we will need better parsers, improved ability to handle passives and negation, the ability to deal with parenthesized text, and further attention to the detection of a variety of semantic classes in addition to genes and proteins. This suggests the necessity of retraining gene mention systems and other taggers on full text, and by extension the importance of building full-text corpora. Future studies will pursue concrete solutions to these issues.

## Methods

### Sentence length

We used the LingPipe sentence segmenter with the MedlineSentenceModel.

### Gold standard for gene mentions

The gold standard for gene mentions was prepared by having an experienced annotator mark up all gene and protein names in the CRAFT corpus, using the BioCreative guidelines.

### Gene mention tagger performance

We used the following tagger versions:

• ABNER [26]: 1.5

• BANNER [27]: 0.2

• LingPipe [28]: 3.1.2

### Mutation mention tagger performance

We used version 1.1 of the MutationFinder application.

### Drug names

As the drug name dictionary, we used the DrugCards information from DrugBank, downloadable at drugbank.ca/downloads. We used the version of drugcards.txt that was available on August 21, 2009, at 4:15 p.m.

We filtered the following things from the file:

• All names containing regular expression characters, i.e. ()[]+.

• All names consisting of amino acids.

• All names consisting of General English words.

• All names less than five characters long.

We required matches against a full word or words, not counting punctuation.

### Disease mentions

We used the BANNER system with a pre-release model for disease names.

### Syntactic parsing

We used version 1.6 of the Stanford Lexicalized Parser.

## Authors' contributions

KBC designed all experiments, ran the parenthesis, mutation, disease, and drug experiments, and wrote the paper. HLJ ran the structural and discourse feature experiments and did the statistics. KV ran one of the drug experiments. CR ran one of the disease experiments. LH supervised all aspects of the work. All five authors reviewed and approved the manuscript.
